# Generation of virus-like particle as a vaccine strategy against bovine leukemia virus

**DOI:** 10.1186/1742-4690-12-S1-P47

**Published:** 2015-08-28

**Authors:** Hiroyuki Otsuki, Shin-nosuke Takeshima, Yoko Aida

**Affiliations:** 1 Viral Infectious Diseases Research Unit, RIKEN, Saitama, Japan

## 

Bovine leukemia virus (BLV) is a causative agent of the enzootic bovine leucosis (EBL) with the increasing rate of infection and incidence in cattle. A vaccine against BLV is needed but individual differences in sensitivity to the disease make it difficult to assess the efficiency of a candidate vaccine. We previously determined that a bovine MHC (BoLA) class II allele correlated to high proviral load and sensitivity to develop lymphoma. Based on the finding, we designed a peptide antigen “Gag p12-4R1” in silico that is a CD4 epitope optimized to fit BoLA-DR of cattle sensitive to EBL and exhibited the peptide had an effect on reducing proviral load of BLV-infected cattle. In this study, we focused on virus-like particle (VLP) vaccine strategy to enhance the antigenicity of the peptide vaccine. Here, we evaluated expression of Gag containing a p12-4R1 epitope and generation of VLP in mammalian cells, insect cells or a silkworm. Wild-type or p12-4R1-mutant gag was cloned under the control of the CMV promoter and they were transfected into mammalian cells. Recombinant baculovirus AcNPV or BmNPV, encoding wild-type or p12-4R1-mutant Gag fused with HA or FLAG-tag, was generated and infected to S.frugiperda cell lines or a silkworm, respectively. As a result, Gag precursor pr44 was highly expressed in all the tested species introduced with wild-type and p12-4R1-mutant gag. Furthermore, VLP formation in mammalian and insect cells was indicated because of detection of pr44 in the pellet of concentrated culture supernatant by ultracentrifugation. VLP vaccine strategy is useful to produce antigens with deleterious mutations such as some mutations in BLV p12 protein that reduce viral RNA incorporation into virion. The physical property of the VLP, its antigenicity in mouse and vaccine efficiency in cattle remain to be assessed in the future study.

